# Complete Genome Sequence of Caldicellulosiruptor changbaiensis CBS-Z, an Extremely Thermophilic, Cellulolytic Bacterium Isolated from a Hot Spring in China

**DOI:** 10.1128/MRA.00021-19

**Published:** 2019-02-28

**Authors:** Carl Mendoza, Sara E. Blumer-Schuette

**Affiliations:** a Department of Biological Sciences, Oakland University, Rochester, Michigan, USA; University of Maryland School of Medicine

## Abstract

Here, we describe the complete genome sequence of Caldicellulosiruptor changbaiensis, isolated from a hot spring in the Changbai Mountain Range of China. Currently, only one other genome sequence representing a *Caldicellulosiruptor* species from China is available. Assembly of a continuous single contig used both Oxford Nanopore and Illumina sequencing data.

## Abstract (precis)

Here, we describe the complete genome sequence of Caldicellulosiruptor changbaiensis, isolated from a hot spring in the Changbai Mountain Range of China. Currently, only one other genome sequence representing a *Caldicellulosiruptor* species from China is available.

## ANNOUNCEMENT

The genus *Caldicellulosiruptor* includes extremely thermophilic, plant biomass-degrading anaerobes, which have been isolated worldwide from terrestrial, geothermally heated springs (1–8) or other thermophilic environments (9, 10). Comparative genomics studies analyzing available genomes (11, 12) have defined the *Caldicellulosiruptor* pangenome, which comprises core genes shared among all species sequenced and dispensable genes found in one or more species (13). Interest in the genus *Caldicellulosiruptor* stems from temperature-stable carbohydrate-active enzymes encoded by the *Caldicellulosiruptor* pangenome that degrade polysaccharide components of plant biomass, such as cellulose and hemicellulose. An open *Caldicellulosiruptor* pangenome (11, 12) indicates the potential for the discovery of additional, as-yet-unsequenced, genes encoding enzymes and proteins of biotechnological use.

Caldicellulosiruptor changbaiensis was isolated from hot spring sediment in the Changbai Mountains of Northeast China (8). An axenic culture of C. changbaiensis CBS-Z (DSM 26941) was obtained from the German Collection of Microorganisms and Cell Cultures (DSMZ). Cells for genomic DNA (gDNA) isolation were revived in low-osmolarity complex (LOC) medium (14) supplemented with cellobiose, incubated anaerobically at 75°C, and harvested at stationary phase. Isolation of gDNA followed an established protocol for bacterial gDNA extraction with the cetyltrimethylammonium bromide (CTAB) method (https://jgi.doe.gov/user-programs/pmo-overview/protocols-sample-preparation-information/). Gel electrophoresis and a Qubit fluorometer were used to assess the DNA quality and concentration.

Long-read sequencing libraries were constructed with a rapid sequencing kit (SQK-RAD004, Oxford Nanopore Technologies) and 400 ng of gDNA per the manufacturer’s instructions. After loading the library into a R9.4.1 flow cell for the MinION platform (FLO-MIN106), sequence data collection proceeded for 22 h. Base calling used Albacore v4.3.2, which resulted in 899,637 passed reads (*N*_50_ length, 7,146 bp), comprising 2,836 Mbp of sequence data. Adapters were trimmed with PoreChop v0.2.4, and sequence data were further analyzed and subsampled with NanoPlot v1.15.0 and NanoFilt v2.2.0 (15) based on a quality score of 11 and a read size greater than 4,000 bp, which resulted in 171 Mbp of data for assembly. Illumina sequencing (HiSeq 2500, 2 × 150 bp) was performed at Molecular Research LP (MR DNA). Libraries were created from 25 ng of gDNA with the KAPA HyperPlus kit (Roche) per the manufacturer’s instructions. A total of 13,695,689 reads passed the quality check (QC) in FastQC v0.11.7 (http://www.bioinformatics.babraham.ac.uk/projects/fastqc/), with an average quality score of 35.9 over all reads.

Hybrid assembly with Unicycler v0.4.7 (16) resulted in a single assembled contig (2,905,633 bp) with 60.5× sequence coverage. Annotation of the C. changbaiensis genome was conducted with the National Center for Biotechnology Information (NCBI) Prokaryotic Genome Annotation Pipeline v4.7 (17). In the 2.9-Mbp genome (G+C content, 35.1%), 2,779 genes were annotated, from which 2,532 genes are predicted to encode proteins. Similar to other sequenced members of the genus *Caldicellulosiruptor*, C. changbaiensis also possesses three loci of 5S, 16S, and 23S rRNA gene sequences (18–21).

### Data availability.

All data associated with this project are available from NCBI (BioProject accession number PRJNA511150), including the assembled contig for C. changbaiensis CBS-Z (GenBank accession number CP034791) and raw sequence reads from Oxford Nanopore and Illumina sequencing (Sequence Read Archive accession numbers SRR8467877 and SRR8467565, respectively).
